# Targeting the NFAT1-MDM2-MDMX Network Inhibits the Proliferation and Invasion of Prostate Cancer Cells, Independent of p53 and Androgen

**DOI:** 10.3389/fphar.2017.00917

**Published:** 2017-12-14

**Authors:** Jiang-Jiang Qin, Xin Li, Wei Wang, Xiaolin Zi, Ruiwen Zhang

**Affiliations:** ^1^Department of Pharmacological and Pharmaceutical Sciences, College of Pharmacy, University of Houston, Houston, TX, United States; ^2^Department of Pharmaceutical Sciences, School of Pharmacy, Texas Tech University Health Sciences Center, Amarillo, TX, United States; ^3^Center for Drug Discovery, University of Houston, Houston, TX, United States; ^4^Department of Urology, University of California, Irvine, Irvine, CA, United States; ^5^Department of Pharmacology, University of California, Irvine, Irvine, CA, United States

**Keywords:** MDM2, MDMX, NFAT1, p53, prostate cancer

## Abstract

The MDM2 and MDMX oncogenes are overexpressed in various types of human cancer and are highly associated with the initiation, progression, metastasis and chemotherapeutic resistance of these diseases, including prostate cancer. The present study was designed to test a natural MDM2 inhibitor, Inulanolide A (InuA), for anti-prostate cancer activity and to determine the underlying mechanism(s) of action. InuA directly bound to the RING domains of both MDM2 and MDMX with high affinity and specificity and disrupted MDM2-MDMX binding, markedly enhancing MDM2 protein degradation. We further discovered that InuA bound to the DNA binding domain of NFAT1, resulting in marked inhibition of *MDM2* transcription. InuA inhibited the proliferation, migration, and invasion of prostate cancer cells, regardless of their p53 status and AR responsiveness. Double knockdown of MDM2 and NFAT1 also revealed that the expression of both of these molecules is important for InuA’s inhibitory effects on the proliferation and invasion of prostate cancer cells. In summary, InuA represents a novel class of bifunctional MDM2 inhibitors, and should be further investigated as a candidate lead compound for prostate cancer prevention and therapy.

## Introduction

Prostate cancer is the most commonly diagnosed cancer and the second leading cause of cancer-related death among men in the United States (Yap et al., 2016). The androgen receptor (AR) is highly expressed in primary and metastatic prostate cancer and regulates multiple cellular functions, including proliferation, apoptosis, migration, invasion, and differentiation in all stages of prostate cancer (Spratt et al., 2016; Leach and Buchanan, 2017). Pharmacological inhibition of AR signaling represents a compelling and conventional therapeutic strategy for prostate cancer, which leads to a >90% 10-year survival of patients with low-risk, localized prostate cancer (Spratt et al., 2016; Leach and Buchanan, 2017). However, many patients eventually develop an incurable, androgen-insensitive phenotype of prostate cancer, termed castration-resistant prostate cancer (CRPC). The benefits obtained with androgen deprivation therapy (ADT) for patients with CRPC are unacceptably low (Yap et al., 2016). Although multiple clinical trials have shown that ADT in combination with radiotherapy can improve the clinical outcomes and survival rates of CRPC patients, the recurrence rates remain high (Falchook et al., 2016; Saad et al., 2017). Therefore, novel therapeutic and preventive approaches for prostate cancer are needed.

The murine double minute (MDM) oncogene family, consisting of MDM2 and MDMX, has been demonstrated to be a promising molecular target for the prevention and treatment of various types of human cancer, including prostate cancer (Burgess et al., 2016; Karni-Schmidt et al., 2016; Lemos et al., 2016). MDM2 and MDMX are well characterized as negative regulators of the tumor suppressor p53 and have been proven to contribute to cancer initiation, progression, and metastasis, as well as the resistance to chemotherapy (Hernandez-Monge et al., 2016; Karni-Schmidt et al., 2016). The binding of the RING domains of MDM2 and MDMX are pivotal for inhibiting p53 (Huang et al., 2011; Pant et al., 2011). The MDM oncoproteins also function as critical regulators of tumor cell proliferation, apoptosis, invasion, and chemoresistance in a p53-independent manner (Karni-Schmidt et al., 2016). Both MDM2 and MDMX are inappropriately upregulated and highly associated with an increased incidence of prostate cancer (Stegeman et al., 2015; Xue et al., 2016). MDM2 also plays a critical role in Akt-mediated AR ubiquitination and protein degradation (Lin et al., 2002). Pharmacological inhibition of MDM2 by small molecule inhibitors (SMIs) results in the inhibition of cell proliferation and induction of apoptosis in prostate cancer cells, as well as sensitization of prostate cancer cells to chemotherapy, radiotherapy, and anti-androgen therapy, regardless of their AR and p53 status (Voruganti et al., 2015a; Feng et al., 2016; Logan et al., 2016). Thus, pharmacological inhibition of the MDM oncoproteins appears to be a promising approach for treating patients with prostate cancer, especially CRPC.

We have previously demonstrated that the NFAT1 transcription factor activates MDM2, independent of p53 (Li et al., 2005; Zhang et al., 2012; Qin et al., 2017a). NFAT family members, including NFAT1, also exert oncogenic functions in prostate cancer (Qin et al., 2014). The expression levels of NFAT isoforms are significantly elevated in prostate cancer and are correlated with an increased risk of recurrence after ADT (Kawahara et al., 2015). NFAT activation promotes cell proliferation and enhances the apoptosis resistance of prostate cancer cells (Thebault et al., 2006; Manda et al., 2016). In addition, activation of calcineurin-NFAT signaling stimulates osteoclastogenesis and inhibits osteoblast apoptosis in prostate cancer, promoting bone metastasis (Rafiei and Komarova, 2013). Importantly, NFAT inhibitors have been found to decrease viability, inhibit migration and invasion, and enhance apoptosis in prostate cancer cells, independent of the AR status (Kawahara et al., 2015).

**FIGURE 1:**
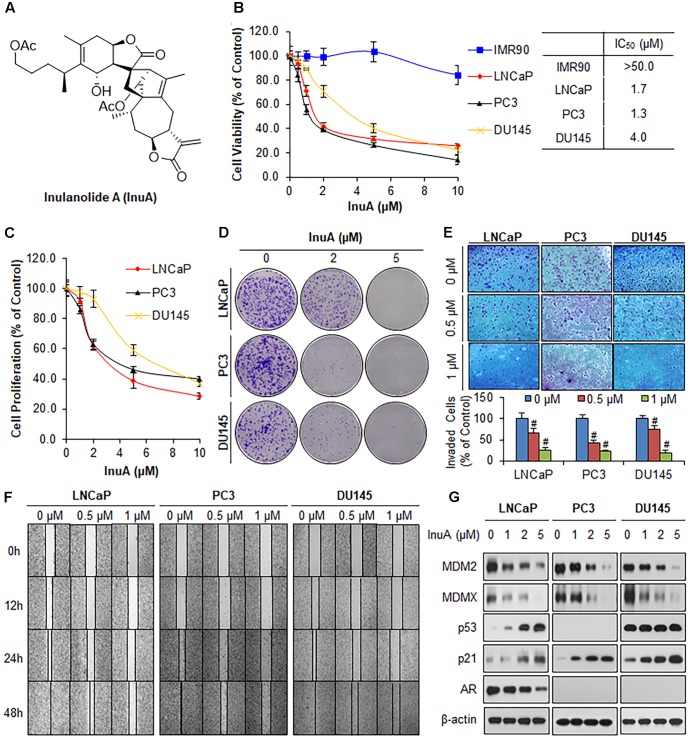
Inulanolide A (InuA) suppresses prostate cancer cell proliferation, migration and invasion, and inhibits the protein expression of MDM2 and MDMX, regardless of the p53 status. **(A)** The chemical structure of InuA. **(B)** IMR90, LNCaP, PC3, and DU145 cells were treated with InuA at the indicated concentrations for 72 h, followed by MTT assays. **(C)** LNCaP, PC3, and DU145 cells were treated with InuA at the indicated concentrations for 24 h, followed by BrdU cell proliferation assays. **(D)** LNCaP, PC3, and DU145 cells were treated with InuA at the indicated concentrations for 24 h, followed by 10-day colony formation assays. **(E)** LNCaP, PC3, and DU145 cells were treated with InuA at the indicated concentrations for 24 h then the cell invasion was determined by transwell invasion assays (^#^*P* < 0.01). **(F)** LNCaP, PC3, and DU145 cells were treated with InuA at the indicated concentrations and cell migration was evaluated by 48-h wound-healing assays. **(G)** LNCaP, PC3, and DU145 were treated with InuA at the indicated concentrations for 24 h, and the levels of various proteins were detected using specific antibodies by Western blotting. The data are representative of three or more experiments.

Because the NFAT1-MDM2 signaling pathway has been implicated in prostate cancer cell proliferation and apoptosis (Li et al., 2005; Zhang et al., 2012), it was hypothesized that targeting the NFAT1–MDM2 interaction could represent a potential therapeutic strategy for prostate cancer. We have recently discovered a novel dimeric sesquiterpenoid, Inulanolide A (InuA, **Figure 1A**), which functions as a dual inhibitor of MDM2 and NFAT1 (Qin et al., 2016b, 2017b). InuA is structurally and functionally distinct from the previously reported SMIs of MDM2 and NFAT1 (Nag et al., 2013; Qin et al., 2014; Lemos et al., 2016). However, the detailed molecular mechanisms of action for this compound have not been fully elucidated, and its impact on MDMX has not been investigated, nor have the molecular binding mechanisms. The present study was designed to investigate the anti-prostate cancer activity of InuA, focusing on its dual-targeting mechanisms of action. Our results demonstrated that InuA has several pharmacological advantages due to its ability to bind MDM2, MDMX, and NFAT1. These results also provided evidence supporting the future development of this bifunctional MDM2 inhibitor for the prevention and treatment of prostate cancer.

## Materials and Methods

### Cell Lines and Cell Culture

Human prostate cancer LNCaP (p53 wild type, AR positive), PC3 (p53 *null*, AR negative) and DU145 (p53 mutant, AR negative) cell lines were purchased from the American Type Culture Collection (Rockville, MD, United States). LNCaP and DU145 cells were cultured in Roswell Park Memorial Institute (RPMI) 1640 medium and PC3 cells were grown in Ham’s F12 medium. Human primary fibroblasts (IMR90) were kindly provided by Dr. S. Lee (Harvard Cutaneous Biology Research Center, Charles Town, MA, United States) and cultured in Dulbecco’s modified Eagle’s medium (DMEM). The MDM2^-/-^ p53^-/-^ and MDMX^-/-^ p53^-/-^ mouse embryonic fibroblast (MEF) cell lines were kind gifts from Dr. G. Lozano (University of Texas, MD Anderson Cancer Center, Houston, TX, United States) and were maintained in DMEM. All cell culture media were supplemented with 10% fetal bovine serum (FBS) and 1% penicillin/streptomycin.

### Chemicals, Antibodies, Plasmids, siRNA, and Other Reagents

InuA was prepared and characterized as described in our previous studies (Qin et al., 2016b). All chemicals and solvents used in the present study were of the highest grade available. The sources of antibodies were as follows: anti-MDM2 (Ab-2; EMD Millipore), anti-MDMX (4194; Bethyl Laboratories), anti-p53 (DO-1; Santa Cruz Biotechnology), anti-p21 (Ab-1; EMD Millipore), anti-NFAT1 (1/NFAT-1; BD Biosciences), anti-NFAT2 (7A6; Santa Cruz Biotechnology), anti-NFAT3 (B-2; Santa Cruz Biotechnology), anti-NFAT4 (F-1; Santa Cruz Biotechnology), anti-NFAT5 (F-9; Santa Cruz Biotechnology), anti-α-Tubulin (B-5-1-2; Sigma), anti-Lamin B (C20; Santa Cruz Biotechnology), anti-β-actin (AC-15; Sigma), anti-GST (GST01; Neomarkers), anti-ubiquitin (6C1; Sigma), anti-Myc (9E10; EMD Millipore), anti-6x-His (HIS.H8; Thermo Fisher Scientific), anti-Flag (M2; Sigma), anti-biotin (BTN.4; Thermo Fisher Scientific), goat anti-mouse IgG (H+L; Bio-Rad), and goat anti-rabbit IgG (H+L; Bio-Rad).

The human *MDM2* P2 promoter reporter was kindly provided by Dr. J. P. Blaydes (Southampton General Hospital, United Kingdom). The GST-MDM2 RING and GST-MDMX RING plasmids were kindly provided by Dr. C. L. Day (University of Otago, Dunedin, New Zealand). His-NFAT1-DBD plasmid was kindly provided by Dr. A. Rao (Harvard Medical School, Boston, MA, United States). The recombinant GST, GST-MDM2 RING, GST-MDMX RING, and His-NFAT1 DBD proteins were prepared and purified as described previously (Linke et al., 2008; Zhang et al., 2012; Wang et al., 2014a). The recombinant His-MDM2 protein was obtained from Abcam (Cambridge, MA, United States). The other vectors used in these studies were generated as reported previously (Qin et al., 2015a,b). The siRNAs against human NFAT1, MDM2, and MDMX were purchased from Thermo Fisher Scientific (Rockford, IL, United States). The transfection of plasmid vectors and siRNAs was performed using the methods described previously (Voruganti et al., 2015b).

### Cell Viability, BrdU Cell Proliferation, Colony Formation, Wound Healing, and Transwell Invasion Assays

Cell viability (Qin et al., 2016b), BrdU cell proliferation (Qin et al., 2016a), colony formation (Wang et al., 2014b), wound healing (Qin et al., 2016a), and transwell invasion (Qin et al., 2016b) assays were performed as described previously. To examine the effects of InuA on cell viability, cells (3000 cells/well) in 96-well plates were treated with the compound at the indicated concentrations for 72 h, followed by a MTT assay. To determine the effects of InuA on cell proliferation, cells (5000 cells/well) in 96-well plates were treated with the compound at the indicated concentrations for 24 h. BrdU was added to the medium 3 h before termination of the experiment. To evaluate the effects of InuA on colony formation, cells (1000 cells/well) in 6-well plates were treated with the compound at the indicated concentrations for 24 h. The treated cells were maintained in fresh medium for another 10 days, followed by fixation and crystal violet staining. To assess the effects of InuA on cell migration, a confluent monolayer of prostate cancer cells was scratched using a pipette tip and exposed to the compound. Each wound was monitored and photographed at 0, 12, 24, and 48 h under a phase-contrast microscope (Olympus America Inc.). To evaluate the effects of InuA on cell invasion, the cells (2.5 × 10^4^ cells/well) were transferred into the upper well of a Boyden chamber and exposed to the compound for 24 h. The cells were then stained with Mayer’s Hematoxylin and Eosin solution and the invading cells were photographed and counted.

### Molecular Modeling

To investigate the binding of InuA-MDM2, InuA-MDMX, and InuA-NFAT1, molecular docking was carried out using the SYBYL-X 2.0 software program (Tripos Associates, Inc., St. Louis, MO, United States). The structure of InuA was constructed using the SYBYL/Sketch module and optimized via Powell’s method by the Tripos force field with the convergence criterion set at 0.05 kcal/(Å mol). The optimized structure of InuA was then assigned to the SYBYL-X 2.0 software using the Gasteiger–Hückel method (Joshi et al., 2016). The X-ray crystal structures of MDM2 (PDB ID: 2VJF), MDMX (PDB ID: 2VJF), and NFAT1 (PDB ID: 1OWR) were imported. An automatic docking mode was used as the protomol generation method (Joshi et al., 2016) while the other docking parameters remained default. Molecular docking studies were performed using the SYBYL/FlexX module, and all results were analyzed using the Pymol 1.7 software^1^.

### Synthesis and Characterization of Biotinylated InuA (Biotin-InuA)

The biotinylated InuA (biotin-InuA) was synthesized and purified to >95% purity before use. The detailed synthetic procedure can be found in the Supplementary Materials and Methods. The spectral data for biotin-InuA are given below:

Biotin-InuA: TLC: (DCM/MeOH, 10:1 v/v): *R_f_* = 0.33; ^1^H NMR (400 MHz, CDCl_3_) δ1.05–1.11 (m, 4H), 1.14 (s, 3H), 1.23–1.25 (m, 2H), 1.30–1.41 (m, 2H), 1.48–1.55 (m, 4H), 1.60–1.65 (m, 5H), 1.76 (s, 3H), 1.87–1.89 (m, 2H), 1.98 (s, 3H), 2.03–2.08 (m, 3H), 2.11–2.14 (m, 4H), 2.14 (s, 3H), 2.20–2.22 (m, 1H), 2.36–2.41 (m, 2H), 2.70–2.75 (m, 2H), 2.82–2.89 (m, 3H), 2.96 (s, 1H), 2.99–3.03 (m, 2H), 3.35 (s, 1H), 3.35–3.41 (m, 3H), 3.93–3.97 (m, 1H), 4.18–4.24 (m, 1H), 4.27 (s, 1H), 4.53 (s, 1H), 4.53–4.73 (m, 2H), 4.94–4.96 (m, 1H), 5.52–5.53 (d, 1H, *J* = 2.8 Hz), 6.21–6.22 (d, 1H, *J* = 3.2 Hz); ^13^C NMR (100 MHz, CDCl_3_) δ14.3, 17.1, 19.7, 20.5, 20.8, 21.2, 26.0, 26.8, 29.8, 31.4, 31.9, 33.8, 34.1, 36.0, 36.5, 36.6, 45.1, 50.8, 54.5, 57.9, 62.6, 64.0, 64.4, 76.0, 81.8, 82.5, 119.3, 130.0, 134.0, 136.5, 137.1, 139.6, 162.6, 170.1, 170.6, 171.0, 178.2; MS (ESI) [M+H]^+^ calculated for C_48_H_66_N_3_O_12_S: 908.43, found: 908.8.

### Streptavidin–Agarose Pulldown Assay

Biotin-InuA and biotin (negative control) were preincubated with streptavidin agarose beads (Invitrogen, Carlsbad, CA, United States) overnight at 4°C. After being washed three times in cold PBS, the biotin-InuA-bound beads were then incubated with the recombinant proteins overnight at 4°C. Non-biotinylated InuA was used as a binding competitor. The bound proteins were separated by SDS–PAGE and immunoblotted with anti-GST, anti-MDM2, or anti-His antibodies.

### Kinetic Studies of the InuA-MDM2 and InuA-MDMX Binding

The specific binding of InuA to MDM2 and MDMX follows the typical covalent binding scheme, where MDM2 or MDMX form an initial encounter complex (InuA:MDM2/MDMX) and then form the irreversible covalently linked complex (InuA-MDM2/MDMX) as follows (Liu et al., 2012; Dong et al., 2015):

$$\text{InuA+MDM2/MDMX}\overset{⇀}{↼}\text{InuA:MDM2/MDMX}\to \text{InuA-MDM2/MDMX}$$

To determine the rate constant for InuA-MDM2/MDMX formation at saturating InuA (*k*_inact_) and the apparent dissociation constant for the initial InuA:MDM2/MDMX complexes (*K*_i_), we incubated MDM2/MDMX with a large excess of biotin-InuA at different time periods and then analyzed the covalently linked InuA-MDM2/MDMX complexes by Western blotting. The band intensities were quantified by the IMAGE J software program. Under the pseudo-first order experimental conditions, the reaction can be shown by the following equations:

$$[\text{MDM}]t={\left[\text{MDM}\right]}_{0}\times {\text{E}}^{{-k}_{obs}\times t}$$

$$\left[\text{MDM-InuA}\right]={\left[\text{MDM}\right]}_{0}-{\left[\text{MDM}\right]}_{t}={\left[\text{MDM}\right]}_{0}\times \left({\text{1-E}}^{{-k}_{obs}\times t}\right)$$

The reaction rate constants of *k*_obs_ at different InuA concentration were obtained by fitting to equation (2), where [MDM]_0_ is the total concentration of MDM2 or MDMX added to the solution and [MDM]*_t_* is the concentration of MDM2 or MDMX at time *t*.

The values of *k*_inact_ and *K*_i_, were determined by fitting *k*_obs_ values to equation (3).

$$k_{obs}=k_{inact}\times [MDM]/\left([MDM]+K_{i}\right)$$

### Western Blotting and Immunoprecipitation Assays

Cell lysates were collected in NP-40 buffer with a protease inhibitor mixture (Sigma–Aldrich, St. Louis, MO, United States). After centrifugation, the supernatants were collected and subjected to a Western blot analysis following the manufacturer’s protocol (Wang et al., 2014a). An immunoprecipitation assay was performed to examine the effects of InuA on the MDM2–MDMX interaction as described previously (Wang et al., 2014b). In brief, the cells were exposed to various concentrations of InuA for 2 h. The cell lysates were then collected and immunoprecipitated with an anti-MDM2 antibody overnight at 4°C. The bound proteins were incubated with protein G-Sepharose beads (Sigma–Aldrich, St. Louis, MO, United States) at 4°C for 2 h and then subjected to Western blotting.

### Electrophoretic Mobility Shift Assay (EMSA) and Chromatin Immunoprecipitation (ChIP) Assay

Cells were treated with InuA and/or ionomycin (ION; an activator of the Ca^2+^-calcineurin-NFAT signaling pathway) for 24 h. The treated cells were extracted using the NE-PER Nuclear and Cytoplasmic Extraction Kit (Thermo Fisher Scientific, Rockford, IL, United States). The nuclear extracts were preincubated with 1 μg of poly-(dI:dC) (Thermo Fisher Scientific, Rockford, IL, United States) and reacted with a biotin-labeled MDM2 probe at room temperature for 30 min. The reaction products were then subjected to EMSA as described previously (Zhang et al., 2012). The sequences of the biotin-labeled probes were: MDM2 forward, 5′-GCAGGTTGACTCAGCTTTTCCTCTTGAGCTGGTCAAGTTCA-3′ and MDM2 reverse, 5′-TGAACTTGACCAGCTCAAGAGGAAAAGCTGAGTCAACCTGC-3′. The ChIP assay has been described previously (Zhang et al., 2012). The sequences of the primer pairs used for qualitative or quantitative PCR amplification of the responsive element of the MDM2 promoter were: 5′-CCCCCGTGACCTTTACCCTG-3′ and 5′-AGCCTTTGTGCGGTTCGTG-3′.

### Real-Time Quantitative PCR and Luciferase Reporter Assay

RNA was extracted from the InuA-treated cells using the Trizol reagent (Invitrogen, Carlsbad, CA, United States) and cDNA was synthesized by SuperScript reverse transcription-PCR (Invitrogen, Carlsbad, CA, United States). The sequences of the primers were as follows: MDM2 sense, 5′-ATCATCGGACTCAGGTACA-3′; MDM2 antisense, 5′-GTCAGCTAAGGAAATTTCAGG-3′; GAPDH sense, 5′-GGAGTCCACTGGCGTCTTCAC-3′; GAPDH antisense, 5′-GAGGCATTGCTGATGATCTTGAGG-3′. The luciferase reporter assay has been described previously (Yang et al., 2012; Zhang et al., 2012). Briefly, the cells co-transfected with the *MDM2* P2 promoter luciferase reporter plasmid and Renilla luciferase reporter were exposed to InuA for 24 h, followed by an examination of the luciferase activity using the Dual-Luciferase Reporter Assay System (Promega, Madison, WI, United States).

### Statistical Analysis

Data are presented as the means ± SEM from three or more independent experiments. Statistical analyses were conducted using the Prism software version 6 (Graph Pad software Inc., San Diego, CA, United States). Student’s *t*-test was used for comparisons between two groups. Values of *P* < 0.05 were considered to be statistically significant.

## Results

### InuA Inhibits Prostate Cancer Cell Growth, Proliferation, Migration, and Invasion, and the Expression of MDM2 and MDMX, Regardless of the p53 and AR Statuses

We first examined the effects of InuA on the growth of three prostate cancer cell lines harboring different p53 and AR backgrounds, including LNCaP (p53 wild type, AR positive), PC3 (p53 *null*, AR negative) and DU145 (p53 mutant, AR negative) cells, and a normal fibroblast cell line (IMR90). As shown in **Figure 1B**, InuA inhibited the growth of all three prostate cancer cell lines with IC_50_ values of 1.7, 1.3, and 4.0 μM, respectively. The IMR90 cells were much less sensitive to the compound than the prostate cancer cell lines, indicating that InuA has a selective cytotoxicity to cancer cells. The anti-proliferative activity of InuA was evaluated by a BrdU cell proliferation assay, which showed a concentration-dependent and p53-independent inhibition of cell proliferation in androgen-sensitive and -resistant prostate cancer cell lines (**Figure 1C**). Similar results were obtained with a colony formation assay, further supporting the inhibitory effects of InuA on prostate cancer cell proliferation (**Figure 1D**).

The compound was further examined for its effects on cell migration and invasion in LNCaP (p53wt, AR positive), PC3 (p53 *null*, AR negative) and DU145 (p53mut, AR negative) cells. In comparison to the DMSO-treated cells, sublethal concentrations of InuA (0.5 and 1 μM) markedly reduced the number of invading cells in a concentration-dependent and p53- and androgen-independent manner (**Figure 1E**). In addition, InuA concentration-dependently prevented prostate cancer cell migration into wounded areas, regardless of the p53 and AR statuses of the cells (**Figure 1F**).

Next, we examined the effects of InuA on the protein expression of MDM2 and MDMX in prostate cancer cells. As shown in **Figure 1G**, InuA decreased the protein expression levels of both MDM2 and MDMX in a concentration-dependent manner in all three prostate cancer cell lines. As the inhibition of MDM2 and MDMX leads to an induction of the p53 and p21 proteins (Jin et al., 2003, 2008; Zhang et al., 2004; Xu et al., 2010), we also examined the ability of InuA to induce p53 and p21. InuA was found to increase the protein expression levels of p21 in all three cell lines and to increase wild type p53 expression in LNCaP cells, but the compound did not affect the expression of mutant p53 in DU145 cells. InuA also inhibited the expression of AR in LNCaP (AR positive) in a concentration-dependent manner (**Figure 1G**).

### InuA Directly Binds to MDM2 and MDMX

**FIGURE 2:**
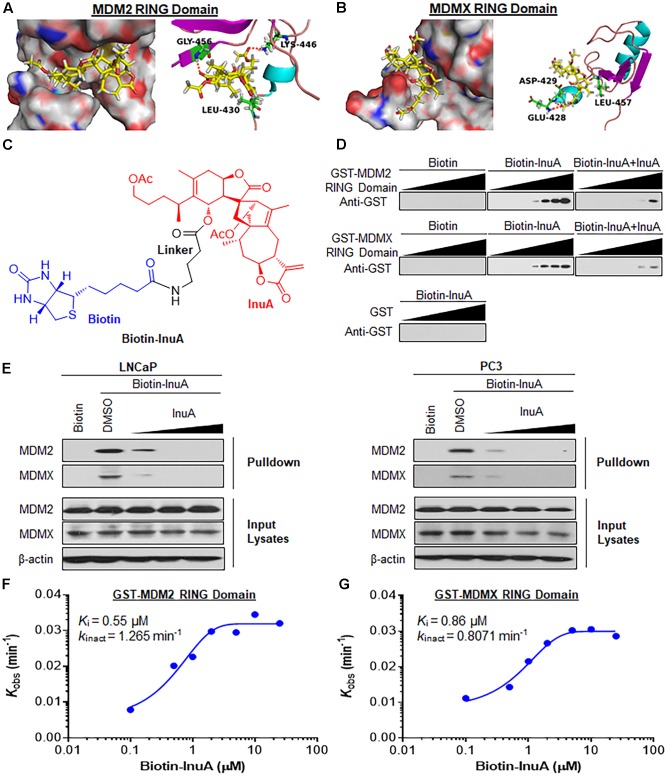
Inulanolide A directly binds to the RING domains of MDM2 and MDMX. **(A,B)** Computational modeling of InuA binding to the RING domains of MDM2 **(A)** and MDMX **(B)**. InuA was rendered in yellow, with the atoms important for binding highlighted in red. The key residues interacting with InuA were rendered as sticks. The hydrogen bonds were shown by red dotted lines. **(C)** The chemical structure of biotinylated InuA (Biotin-InuA). **(D)** Biotin-InuA-conjugated avidin beads were incubated with various concentrations of purified GST-MDM2 RING or GST-MDMX RING in the presence or absence of non-biotinylated InuA. Purified GST was used as a negative control. The bound proteins were detected using anti-GST antibodies. **(E)** Biotin-InuA-conjugated avidin beads were incubated with LNCaP and PC3 cell lysates in the presence or absence of a 2-, 10-, or 20-fold excess of non-biotinylated InuA. The mixtures were blotted for bound MDM2 and MDMX proteins. **(F,G)** Plots of the *k*_obs_ values for the binding of the MDM2 RING domain **(F)** and the MDMX RING domain **(G)** as a function of the InuA concentration. Data are representative of three or more experiments.

InuA is a dimeric sesquiterpenoid which possesses a conformationally flexible scaffold. The compound bears an α-methylene-γ-lactone moiety, which functions as a reactive Michael acceptor and may form a covalent bond with the MDM2 and MDMX proteins. Based on our molecular docking studies, InuA specifically bound to the C-terminal RING domains of MDM2 and MDMX via interactions with LEU430, LYS446, and GLY456 in the MDM2 protein (**Figure 2A**) and with GLU428, ASP429, and LEU457 in the MDMX protein (**Figure 2B**). The carbonyl group next to the spiro-atom in InuA directly interacted with GLY456 in MDM2 via a hydrogen bond (**Figure 2A**). The acetyl group attached to the long alkyl chain in InuA formed a hydrogen bond with LYS446 in MDM2 (**Figure 2A**) and with GLU428 and ASP429 in MDMX (**Figure 2B**). In addition, the methyl group in the alkyl chain also displayed a hydrophobic interaction with LEU430 in MDM2 (**Figure 2A**) and LEU457 in MDMX (**Figure 2B**).

To confirm whether InuA directly binds to MDM2 and MDMX, we synthesized biotinylated InuA (biotin-InuA) (**Figure 2C**) and prepared recombinant GST, GST-MDM2 RING, and GST-MDMX RING proteins. Pulldown assays using these constructs validated that InuA directly bound to the MDM2 RING and MDMX RING proteins, but not the GST tag (**Figure 2D**). The formation of the InuA-MDM2 RING and InuA-MDMX RING complexes was significantly decreased by the addition of competitive non-biotinylated InuA (**Figure 2D**). Similar results were obtained with LNCaP and PC3 whole cell lysates (**Figure 2E**). There was also a dose-dependent inhibition of the InuA-MDM2 and InuA-MDMX binding by the non-biotinylated InuA (**Figure 2E**).

### Kinetics of InuA-MDM2 and InuA-MDMX Binding

We next investigated the binding kinetics of the InuA-MDM2 and InuA-MDMX complexes. We examined the extent of MDM2 and MDMX binding at several time points for various biotin-InuA concentrations. The progress of MDM2 and MDMX binding with InuA at different concentrations showed time-dependent saturation (Supplementary Figure 1), suggesting an irreversible mechanism of binding (Liu et al., 2012; Dong et al., 2015). The results were fit to various models to determine the observed rate constants for binding (*k*_obs_) at each concentration of the compound (Supplementary Figure 1). Further plotting these *k*_obs_ values as a function of the biotin-InuA concentration resulted in respective saturation curves for InuA-MDM2 (**Figure 2F**) and InuA-MDMX (**Figure 2G**) binding. These results suggested that there was a two-step reaction mechanism for the inactivation of MDM2 and MDMX by InuA. The initial binding step (*K*_i_), i.e., the non-covalent reversible binding of InuA to MDM2 and MDMX, placed the moderately reactive electrophile of InuA close to a specific nucleophile on the proteins. In the second chemical step, the formation of the specific covalent linkage (*k*_inact_) resulted in the inhibited MDM2-MDMX complex. On the basis of this model, the *k*_inact_ values for the binding of InuA to MDM2 and MDMX were determined to be 1.265 and 0.8071 min^-1^, respectively. The *K*_i_ values for InuA-MDM2 and InuA-MDMX binding were 0.55 and 0.86 μM, respectively. Taken together, the findings indicated that InuA effectively bound to both MDM2 and MDMX (*k*_inact_/*K*_i_ = 3.83 × 10^4^ M^-1^s^-1^ and 1.56 × 10^4^ M^-1^s^-1^, respectively), with high affinity and specificity. These kinetic data also demonstrated that InuA bound to MDM2 more potently than it bound to MDMX.

### InuA Disrupts the MDM2–MDMX Interaction and Induces Rapid Degradation of MDM2 and MDMX

**FIGURE 3:**
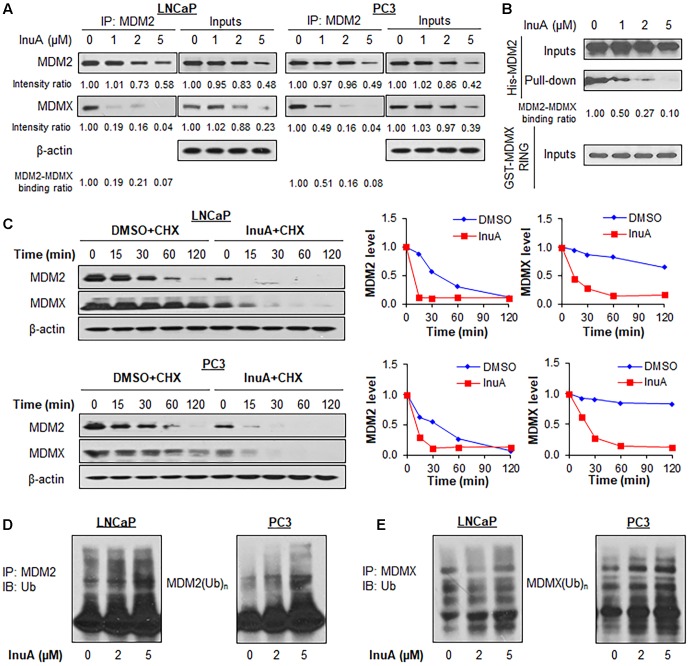
Inulanolide A disrupts the MDM2–MDMX interaction and induces the ubiquitination and degradation of MDM2 and MDMX. **(A)** LNCaP and PC3 cells were treated with InuA at the indicated concentrations for 2 h, followed by the co-immunoprecipitation of the MDM2-MDMX complex with an MDM2 antibody. The protein levels of MDM2 and MDMX were determined by Western blotting. Band intensity ratios were obtained using the IMAGE J software program, and were normalized to those of vehicle controls, and the binding ratios were also normalized to the intensity of the input. **(B)** The His-MDM2 and GST-MDMX RING domain proteins were incubated with InuA at the indicated concentrations overnight at 4°C. The complexes were immobilized on glutathione-sepharose beads for 2 h at 4°C and blotted for the binding of His-MDM2 to GST-MDMX RING using anti-His antibody. **(C)** LNCaP and PC3 cells were treated with InuA (2 μM) for 24 h, followed by exposure to a protein synthesis inhibitor, cycloheximide (CHX, 15 μg/mL). The protein expression levels of MDM2 and MDMX were detected by Western blotting at indicated times after exposure to CHX. Graphs (on the right) show the quantification of the immunoblotting data. **(D)** LNCaP and PC3 cells were co-transfected with MDM2 and ubiquitin plasmids, followed by treatment with InuA at the indicated concentrations for 24 h. Cell lysates were subjected to immunoprecipitation with an MDM2 antibody. The ubiquitinated MDM2 was detected using an anti-ubiquitin antibody. **(E)** LNCaP and PC3 cells were co-transfected with MDMX and ubiquitin plasmids, followed by treatment with InuA at the indicated concentrations for 24 h. Cell lysates were subjected to immunoprecipitation with an MDMX antibody. The ubiquitinated MDMX was detected using an anti-ubiquitin antibody. Data are representative of at least three experiments.

The formation of the MDM2/MDMX RING domain heterodimer stabilizes the MDM2 protein, whereas disruption of MDM2-MDMX binding enhances MDM2 auto-ubiquitination and degradation (Tanimura et al., 1999; Linke et al., 2008). We therefore hypothesized that the specific binding of InuA to the RING domains of MDM2 and MDMX could inhibit the MDM2-MDMX complex, accelerating MDM2 auto-degradation. To test this hypothesis, an immunoprecipitation assay was performed to examine whether InuA affects the MDM2-MDMX complex in prostate cancer cells. As shown in **Figure 3A**, after a 2-h treatment with InuA, the MDM2 expression was reduced only by the higher concentrations of InuA (2 or 5 μM), while the MDM2-MDMX binding was largely inhibited beginning at the effective dose of 1 μM. We further incubated recombinant His-MDM2 and GST-MDMX RING domain proteins with InuA at various concentrations. InuA eliminated the MDM2-MDMX binding in a concentration-dependent manner (**Figure 3B**).

To determine whether the InuA-induced disruption of the MDM2-MDMX complex destabilizes the MDM2 protein, we examined InuA’s effects on the turnover of the MDM2 protein in LNCaP and PC3 cell lines. As shown in **Figure 3C**, InuA shortened the half-life of MDM2 and promoted its more rapid degradation in both cell lines. Surprisingly, the compound also strongly shortened the half-life of MDMX. To determine if InuA destabilizes MDM2 by enhancing its ubiquitination, both LNCaP and PC3 cells were transfected with MDM2 and ubiquitin plasmids, followed by exposure to InuA. As shown in **Figure 3D**, InuA enhanced MDM2 ubiquitination in both cell lines. InuA treatment also induced MDMX ubiquitination in prostate cancer cells (**Figure 3E**).

### MDMX Knockdown Reduces MDM2 Ubiquitination by InuA

**FIGURE 4:**
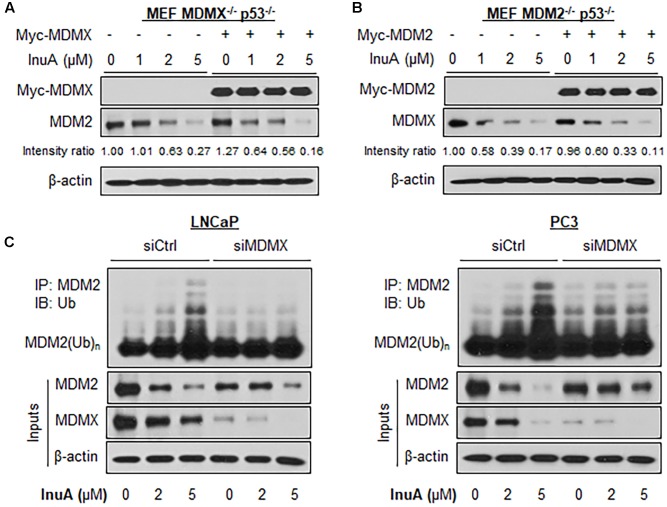
MDMX is important for the InuA-induced MDM2 degradation. **(A)** MEF MDMX^-/-^ p53^-/-^ cells were transfected with a Myc-MDMX plasmid or the empty vector for 24 h, followed by a 24-h exposure to InuA at the indicated concentrations. The expression levels of Myc-MDMX and MDM2 were determined by a Western blot analysis. **(B)** MEF MDM2^-/-^ p53^-/-^ cells were transfected with a Myc-MDM2 plasmid or the empty vector for 24 h, followed by a 24-h exposure to InuA at the indicated concentrations. The expression levels of Myc-MDM2 and MDMX were determined by Western blotting. Band intensity ratios were obtained using the IMAGE J software program and were normalized to those of vehicle controls. **(C)** LNCaP and PC3 cells were transfected with MDMX siRNA (siMDMX) or the respective control siRNA (siCtrl) for 36 h, followed by exposure to InuA at the indicated concentrations for 24 h. Cell lysates were subjected to immunoprecipitation with an MDM2 antibody. The ubiquitinated MDM2 was detected using an anti-ubiquitin antibody. Data are representative of three or more experiments.

Previous studies have discovered that MDMX overexpression stabilizes MDM2 (Tanimura et al., 1999) whereas MDMX knockdown reduces MDM2 expression (Gu et al., 2002). To gain further understanding of the role of MDMX in InuA’s inhibitory effects on MDM2, we transfected MDMX^-/-^ p53^-/-^ MEF cells with a Myc-MDMX plasmid then treated the transfected cells with InuA. As shown in **Figure 4A**, enforced expression of MDMX enhanced the InuA-induced MDM2 degradation in MDMX^-/-^ p53^-/-^ MEF cells at 1 μM. In comparison with the respective control, MDMX overexpression showed moderate enhancement on the MDM2 degradation by InuA at 2 and 5 μM, which suggested that other signaling pathways might also be involved in MDM2 inhibition by InuA at higher concentrations. Conversely, overexpression of MDM2 had no marked effect on the InuA-induced MDMX degradation in MDM2^-/-^ p53^-/-^ MEF cells (**Figure 4B**).

Next, both LNCaP and PC3 cells were transfected with a siRNA targeting MDMX or with non-specific siRNA, followed by exposure to InuA. As shown in **Figure 4C**, InuA enhanced MDM2 ubiquitination and inhibited the protein expression of MDM2 and MDMX in both LNCaP and PC3 cells transfected with non-specific siRNA. However, MDMX KD blocked the InuA-induced MDM2 ubiquitination and reduced InuA’s inhibitory effects on MDM2 expression. However, at the 5 μM concentration, InuA still reduced the MDM2 expression level in MDMX KD cells, indicating that InuA might also inhibit MDM2 via other signaling pathways at higher concentrations.

### InuA Inhibits NFAT1-Mediated *MDM2* Transcription

**FIGURE 5:**
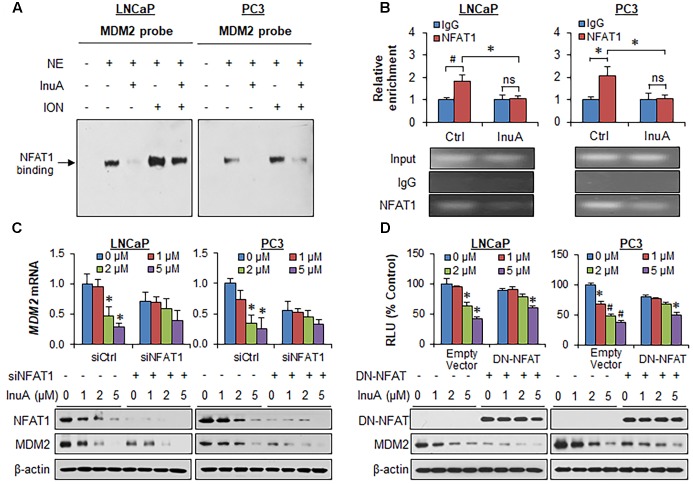
Inulanolide A inhibits NFAT1-mediated *MDM2* transcription. **(A)** LNCaP and PC3 cells were exposed to InuA (5 μM) for 24 h in the presence or absence of ionomycin (ION). Nuclear proteins were extracted and incubated with an MDM2 probe, followed by an EMSA assay. NE, nuclear extract. **(B)** LNCaP and PC3 cells were exposed to InuA (5 μM) for 24 h. The crosslinked chromatin was immunoprecipitated with anti-NFAT1 or IgG antibodies, followed by a real-time PCR analysis. **(C)** LNCaP and PC3 cells were transfected with NFAT1 siRNA or the respective control siRNA for 36 h, followed by exposure to InuA at the indicated concentrations for 24 h. The relative *MDM2* mRNA levels and the protein levels of NFAT1 and MDM2 were determined by quantitative real-time PCR and Western blotting, respectively. **(D)** LNCaP and PC3 cells were co-transfected with DN-NFAT and *MDM2* P2 promoter luciferase for 24 h, followed by exposure to InuA at the indicated concentrations for 24 h. The relative luciferase levels were then determined using a dual-reporter gene detection system. The protein levels of DN-NFAT and MDM2 were determined by a Western blot analysis. Data are representative of at least three experiments (^∗^*P* < 0.05, ^#^*P* < 0.01, and ns, not significant).

It has previously been observed that InuA inhibits the protein expression of an MDM2 activator, NFAT1 (Qin et al., 2016b), which directly binds to the *MDM2* P2 promoter via its DNA binding domain (DBD) and activates *MDM2* transcription (Zhang et al., 2012). To determine whether InuA inhibits the NFAT1-*MDM2* P2 promoter complex, we carried out an EMSA assay and a ChIP assay. As shown in **Figure 5A**, InuA inhibited the specific binding of endogenous NFAT1 to the *MDM2* probe. Our previous studies have shown that ionomycin (ION), an activator of the calcineurin-NFAT pathway can recruit NFAT1 to the *MDM2* P2 promoter, enhancing *MDM2* transcription (Zhang et al., 2012). We further discovered that InuA also significantly inhibited the ION-enhanced NFAT1-*MDM2* P2 promoter binding (**Figure 5A**). The specific inhibition of the NFAT1-*MDM2* P2 promoter binding by InuA was confirmed by a ChIP assay (**Figure 5B**).

To assess whether the disruption of the NFAT1-*MDM2* P2 promoter complex by InuA could inhibit *MDM2* transcription, we examined the *MDM2* mRNA expression and the protein expression of NFAT1 and MDM2 in prostate cancer cells transfected with NFAT1 siRNA or control siRNA. InuA efficiently downregulated *MDM2* mRNA expression, whereas NFAT1 KD markedly decreased the basal *MDM2* mRNA levels and reduced the inhibitory effects of InuA on *MDM2* mRNA expression (**Figure 5C**). InuA also strongly inhibited the protein expression of NFAT1 in both LNCaP and PC3 cells (**Figure 5C**). InuA was further assessed for its inhibitory effects on *MDM2* P2 promoter activity in prostate cancer cells transfected with dominant-negative NFAT (DN-NFAT). As expected, the lower concentrations of InuA (1 or 2 μM) exhibited significant inhibitory effects on *MDM2* P2 promoter activity in the cells transfected with the empty vector. However, only a higher concentration (i.e., 5 μM) of InuA showed marked activity in DN-NFAT-expressing cells (**Figure 5D**).

### InuA Specifically Targets the DNA Binding Domain (DBD) on NFAT1

**FIGURE 6:**
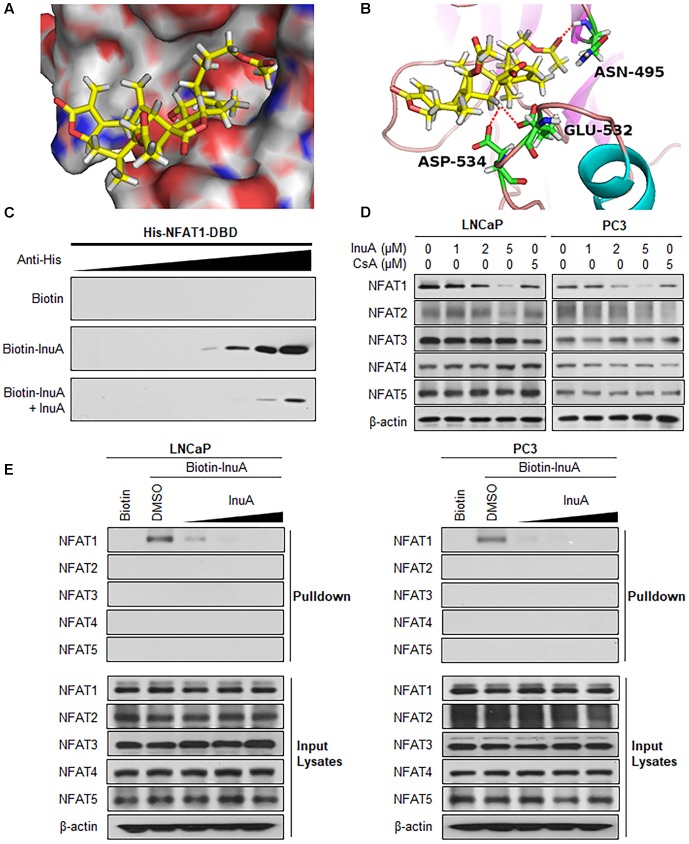
Inulanolide A directly binds to the NFAT1 DNA binding domain. **(A)** Computational modeling of the binding of InuA to the DNA binding domain (DBD) of NFAT1. InuA was rendered in yellow, with the atoms important for binding highlighted in red. **(B)** The predicted binding mode of InuA with the NFAT1 DBD. The key residues interacting with InuA were rendered as sticks. The hydrogen bonds were shown by red dotted lines. **(C)** Biotin-InuA-conjugated avidin beads were incubated with various concentrations of purified His-NFAT1 DBD in the presence or absence of non-biotinylated InuA. The bound protein was detected using an anti-His antibody. **(D)** LNCaP, PC3, and DU145 cells were treated with InuA at the indicated concentrations or with cyclosporine A (CsA; 2 μM) for 24 h, and the levels of NFAT proteins were detected by Western blotting using specific antibodies. **(E)** Biotin-InuA-conjugated avidin beads were incubated with LNCaP and PC3 cell lysates in the presence or absence of a 2-, 10-, and 20-fold excess of non-biotinylated InuA. The mixtures were blotted for bound NFAT proteins.

Next, we examined whether InuA could bind to the NFAT1 protein. According to a molecular docking study, InuA directly bound to the NFAT1 DBD (**Figure 6A**). The InuA-NFAT1 binding model (**Figure 6B**) showed that the acetyl group attached to the long alkyl chain in InuA directly interacted with ASN495 via a hydrogen bond, while the hydroxyl group of InuA displayed hydrogen bond interactions with GLU532 and ASP534 in the DBD of NFAT1. Pulldown assays showed that biotin-InuA, but not biotin alone, specifically bound to the NFAT1 DBD, and this binding was significantly reduced by non-biotinylated InuA (**Figure 6C**).

Because there are five NFAT isoforms, NFAT1–NFAT5, which have structural similarity, we examined the effects of InuA on all five NFAT isoforms. As shown in **Figure 6D**, InuA had a strong inhibitory effect on the expression of NFAT1. The compound also had an inhibitory effect on NFAT2 expression at the higher concentration (5 μM). However, there were no significant effects on the expression of the other NFAT isoforms. Cyclosporine A (CsA), which directly binds to immunophilin and inhibits calcineurin activity, is used a classical inhibitor of the calcineurin-NFAT signaling axis (Qin et al., 2014). In this study, CsA was used as a positive control and showed inhibitory effects on the expression of the calcineurin-responsive NFAT isoforms NFAT1–4. However, it did not significantly affect NFAT5, which has a distinct domain structure. Based on these observations, InuA specifically bound to the NFAT1 protein, but not to other NFAT isoforms, in prostate cancer cell lysates. The InuA-NFAT1 binding was inhibited by the addition of the non-biotinylated InuA (**Figure 6E**).

### InuA Promotes NFAT1 Protein Degradation and Inhibits NFAT1 Nuclear Translocation

**FIGURE 7:**
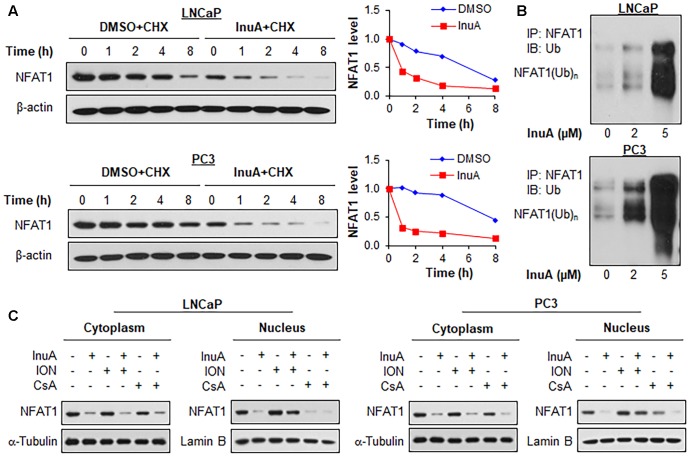
Inulanolide A promotes NFAT1 protein degradation and inhibits NFAT1 nuclear translation. **(A)** LNCaP and PC3 cells were treated with InuA (2 μM) for 24 h, followed by exposure to a protein synthesis inhibitor, cycloheximide (CHX, 15 μg/mL). The protein expression levels of NFAT1 were detected by Western blotting at the indicated times after exposure to CHX. Graphs (on the right) show the quantification of the immunoblotting data. **(B)** LNCaP and PC3 cells were co-transfected with NFAT1 and ubiquitin plasmids, followed by treatment with InuA at the indicated concentrations for 24 h. Cell lysates were subjected to immunoprecipitation with an anti-NFAT1 antibody. The ubiquitinated NFAT1 was detected using an anti-ubiquitin antibody. **(C)** LNCaP and PC3 cells were treated with InuA (2 μM) in the presence or absence of ionomycin (ION; 4 μM) or cyclosporine A (CsA; 2 μM) for 24 h. The nuclear and cytosolic proteins were extracted and examined by Western blotting. Lamin B and α-tubulin were, respectively, used as the internal references for the nuclear and cytosolic extracts. Data are representative of three or more experiments.

We examined the effects of InuA on the protein stability of NFAT1. As shown in **Figure 7A**, InuA shortened the half-life of NFAT1 and enhanced its protein degradation in both LNCaP and PC3 cells. Further studies showed that InuA promoted NFAT1 ubiquitination in a concentration-dependent manner in both cell lines (**Figure 7B**). Next, we examined the effects of InuA on the NFAT1 localization in prostate cancer cells. As shown in **Figure 7C**, InuA reduced the protein levels of both cytoplasmic and nuclear NFAT1. We also examined the effects of InuA on INO-induced NFAT1 nuclear import and CsA-induced NFAT1 nuclear export. It was observed that InuA inhibited the increased nuclear NFAT1 by ION, but could not enhance the inhibitory effects of CsA on nuclear NFAT1 (**Figure 7C**).

### The Expression of Both MDM2 and NFAT1 Is Important for InuA’s Anticancer Activity

**FIGURE 8:**
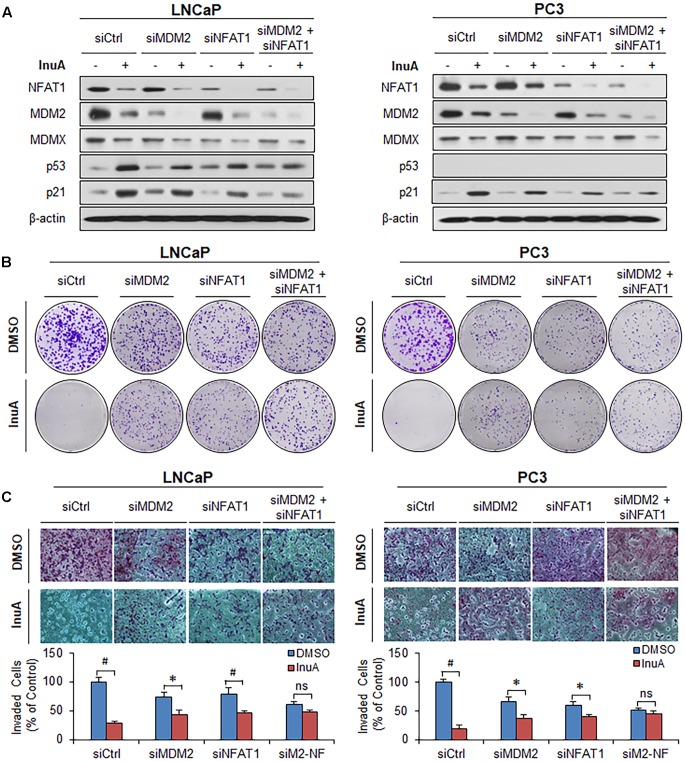
MDM2 and NFAT1 play critical roles in the anticancer activity of InuA. **(A–C)** LNCaP and PC3 cells were transfected with MDM2, NFAT1 or control siRNA for 36 h. The transfected cells were treated with InuA at the indicated concentrations for 24 h for **(A)** an assessment of the expression levels of various proteins by Western blotting, **(B)** the colony formation assay, or **(C)** a transwell invasion assay. Data are representative of at least three experiments. (^∗^*P* < 0.05, ^#^*P* < 0.01, and ns, not significant).

In order to assess the roles of MDM2 and NFAT1 in InuA’s anticancer activity, we transfected the LNCaP and PC3 cells with siRNAs targeting MDM2, NFAT1, or both, as well as with a control siRNA. As shown in **Figure 8A**, single KD of either MDM2 or NFAT1 reduced the InuA-induced expression of p53 and p21. Double KD of MDM2 and NFAT1 almost completely blocked InuA’s effects on the expression of p53 and p21. However, the silencing of MDM2 and NFAT1 did not markedly affect InuA’s inhibitory effects on MDMX. Further, the single KD of MDM2 or NFAT1 largely reduced InuA’s inhibition of colony formation (**Figure 8B**) and cell invasion (**Figure 8C**) in both LNCaP and PC3 cells. The double KD of MDM2 and NFAT1 expression in both LNCaP and PC3 cells completely blocked the inhibitory effects of InuA on cell proliferation and invasion (**Figures 8B,C**).

## Discussion

Novel approaches for inhibiting the MDM oncoproteins, MDM2 and MDMX, are worthy of investigation and might be used clinically for the prevention and treatment of human prostate cancer, especially CRPC. In the present study, we assessed the anticancer activity of a novel bifunctional MDM2 inhibitor, InuA, and investigated its dual mechanisms of action in human prostate cancer models.

We have shown that InuA exerts robust inhibitory effects on the proliferation, migration, and invasion of prostate cancer cells, as well as on the protein expression of MDM2 and MDMX, in a p53- and androgen-independent manner. Because MDMX interacts with MDM2 through its C-terminal RING domain and inhibits the auto-ubiquitination and degradation of MDM2, inhibiting the MDM2–MDMX interaction and MDMX expression may destabilize the MDM2 protein (Tanimura et al., 1999; Linke et al., 2008). In the present study, we demonstrated that InuA directly bound to the RING domains of MDM2 and MDMX and inhibited the binding of MDM2 to MDMX, resulting in enhanced MDM2 auto-ubiquitination and proteasomal degradation, regardless of the p53 status of the cells. Because MDMX protein was also destabilized in response to InuA treatment in prostate cancer cells, it is at least partially responsible for the InuA-induced MDM2 destabilization. However, the results from the MDMX KD prostate cancer cells not only validated the importance of MDMX for InuA-induced MDM2 degradation, but also implied the existence of other molecular mechanisms, such as inhibition of *MDM2* transcription, that contribute to InuA’s inhibitory effects on MDM2.

Consistent with previous studies (Zhang et al., 2012), we observed that the endogenously expressed NFAT1 protein from the nucleus of prostate cancer cells directly bound to *MDM2* DNA and activated MDM2 expression, as evidenced by both EMSA and ChIP assays. NFAT1 is overexpressed and/or constitutively activated in several types of human cancer, and has been implicated in cancer development and progression (Qin et al., 2014). Because InuA exhibited a concentration-dependent inhibition of NFAT1 in prostate cancer cells, we examined the role of NFAT1 in InuA’s anti-MDM2 and anticancer activities. We found that InuA specifically bound to the DBD of NFAT1 and disrupted its binding to the *MDM2* P2 promoter, subsequently inhibiting NFAT1-mediated *MDM2* transcription, independent of p53. Further, NFAT1 KD significantly reduced InuA’s inhibitory effects on *MDM2* mRNA expression and *MDM2* P2 promoter activity, suggesting that the effects of InuA on NFAT1 were associated with its inhibitory effects on *MDM2* transcription. We also found that InuA induced NFAT1 ubiquitination and degradation and inhibited NFAT1 nuclear expression. Finally, we found that the inhibitory effects of InuA on MDM2 and NFAT1 were critical for its anti-prostate cancer activities, as demonstrated using MDM2 and NFAT1 double KD prostate cancer cell lines. Therefore, the dual inhibitory effects of InuA on the MDM2–MDMX interaction and the NFAT1–*MDM2* interaction may translate into enhanced anti-prostate cancer activity.

There remain unanswered questions about the potential use of InuA for prostate cancer. Although we demonstrated that InuA directly bound to the MDM2, MDMX and NFAT1 proteins, we have not yet determined the critical amino acid residues for the binding interactions, which might shed light on future design and development of more specific dual inhibitors of MDM2/MDMX and NFAT1. The binding affinity and kinetics of InuA to its targets need to be further examined using more advanced methods, such as surface plasmon resonance (SPR) and isothermal titration calorimetry (ITC). The molecular mechanisms underlying InuA’s inhibitory effects on MDMX and NFAT1 have yet to be determined. Moreover, there is no direct evidence showing a role for MDMX in the anti-prostate cancer activity of InuA. Of note, NFAT1 is an important regulator of immune responses (Qin et al., 2014), and the potential effects of InuA on the immune system should be investigated in future studies. Although the *in vivo* efficacy of InuA has been demonstrated using an orthotopic tumor model (Qin et al., 2016b), the compound should be further evaluated in clinically relevant models of human prostate cancer, e.g., transgenic prostate cancer models and patient-derived xenograft models, to elucidate its efficacy and safety profile.

In summary, InuA is a novel bifunctional MDM2 inhibitor with high binding affinity to MDM2, MDMX, and NFAT1. The unique molecular mechanisms of action of InuA offer higher potency against MDM2 and stronger anticancer efficacy than any of the previously reported MDM2 and NFAT1 inhibitors. Taken together, our results indicate that dual inhibition of the MDM2–MDMX interaction and the NFAT1–MDM2 interaction might serve as a compelling approach for improving the clinical outcomes of patients with cancer. InuA might be a promising drug candidate for the prevention and treatment of high-risk prostate cancer, especially CRPC.

## Author Contributions

J-JQ and XL designed and conducted experiments, and wrote the manuscript. XZ helped study design and interpretation of data.WW and RZ organized, conceived, and supervised the study. All authors read and approved the manuscript.

## Conflict of Interest Statement

The authors declare that the research was conducted in the absence of any commercial or financial relationships that could be construed as a potential conflict of interest.

## Footnotes

1 http://www.pymol.org/
