# Supplementary material for: Targeting the NFAT1-MDM2-MDMX Network Inhibits the Proliferation and Invasion of Prostate Cancer Cells, Independent of p53 and Androgen
Source: Front Pharmacol. 2017 Dec 14;8:917. doi: 10.3389/fphar.2017.00917 (PMC5735069; doi:10.3389/fphar.2017.00917)
Supplement: Supplementary file 1 [file Image_1.pdf]

Supplementary Information

Targeting the NFAT1-MDM2-MDMX Network Inhibits the Proliferation and Invasion of Prostate Cancer Cells, Independent of p53 and Androgen

Jiang-Jiang Qin[1,2], Xin Li[1,2], Wei Wang[1,2,3], Xiaolin Zi[4,5] and Ruiwen Zhang[1,2,3*]

Authors' Affiliations:

[1] *Department of Pharmacological and Pharmaceutical Sciences, College of Pharmacy, University of Houston, Houston, Texas 77204, USA*

[2] *Department of Pharmaceutical Sciences, School of Pharmacy, Texas Tech University Health Sciences Center, Amarillo, Texas 79106, USA*

[3] *Center for Drug Discovery, University of Houston, Houston, Texas 77204, USA*

[4] *Department of Urology, University of California, Irvine, Irvine, CA, United States,*

[5] *Department of Pharmacology, University of California, Irvine, Irvine, CA, United States*

SUPPLEMENTARY FIGURES

Supplementary Figure 1. (**A**) The determination of the k_{obs} values for the binding of biotin-InuA at various concentrations with MDM2 RING at different times. (**B**) The determination of the k_{obs} values for the binding of biotin-InuA at various concentrations with MDMX RING at different times.

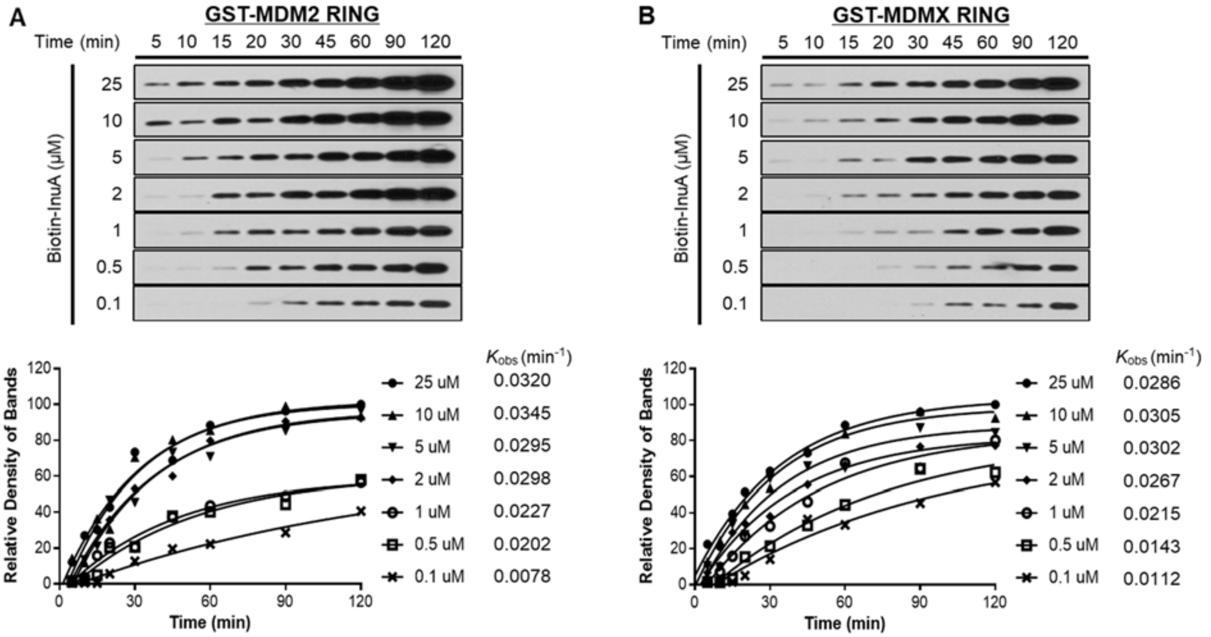

SUPPLEMENTARY MATERIALS AND METHODS

Synthesis and Characterization of Biotinylated InuA (Biotin-InuA)

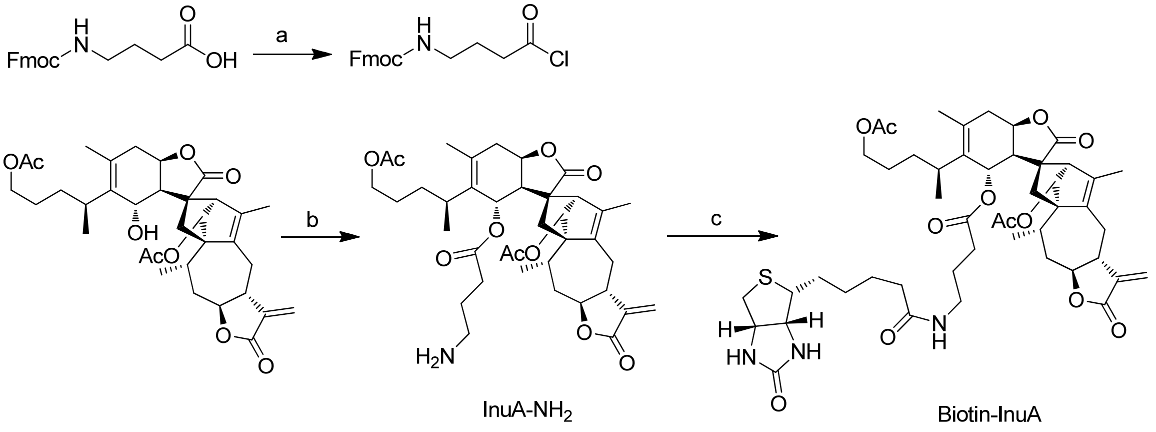

Synthetic route for Biotin-InuA. Regents and Conditions: (a) Oxalyl chloride, DMF, THF, 20 min; (b) (9*H*-fluoren-9-yl)methyl (4-chloro-4-oxobutyl)carbamate, TEA, DMF, overnight; (c) Biotin, EDCI, HOBt, TEA, DMF, overnight.

The 4-((((9H-fluoren-9-yl)methoxy)carbonyl)amino)butanoic acid (11 mg, 0.03 mmol) was dissolved in dry THF (0.5 mL), and DMF (5 μL) was added with stirring. Subsequently, oxalyl chloride (13 mg, 0.10 mmol) was added slowly to the above solution at 0ºC. The mixture was stirred for 20 minutes and then evaporated to dryness by vacuum distillation. The crude product ((9H-fluoren-9-yl)methyl(4-chloro-4-oxobutyl)carbamate) was obtained without further purification.

InuA (20 mg, 0.03 mmol) was dissolved in dry DMF (0.2 mL) and TEA (10 mg, 0.1 mmol) was added with stirring. The crude (9H-fluoren-9-yl)methyl(4-chloro-4-oxobutyl)carbamate was dissolved in dry DMF (0.2 mL) and then added to the above solution at 0ºC. The reaction mixture was slowly warmed to room temperature and stirred overnight. Distilled water (5 mL) was added to quench the reaction. The aqueous layer was extracted with dichloromethane (4 × 5 mL), and the organic layer was washed with a saturated $NaHCO_3$ solution (2 × 3 mL) and a saturated NaCl solution (2 × 3 mL). Subsequently, the organic layer was dried over anhydrous Na_2SO_4 and filtered. The filtrate was concentrated under vacuum to afford the corresponding crude intermediate (InuA-NH_2) without further purification (TLC: DCM/MeOH, 30:1 v/v, R_f = 0.72).

Biotin (15 mg, 0.06 mmol), EDCI (12 mg, 0.06 mmol) and HOBt (8 mg, 0.06 mmol) were dissolved in dry DMF (0.8 mL), then TEA (6 mg, 0.06 mmol) was added with stirring for 1 hour. The crude InuA-NH2 was dissolved in dry DMF (0.2 mL) and then slowly added to the above solution. The mixture was stirred overnight at room temperature. The reaction was quenched with distilled water (6 mL), and the aqueous layer was extracted with dichloromethane (4 × 5 mL). The combined organic layer was washed with saturated $NaHCO_3$ solution (2 × 3 mL), saturated NaH_2PO_4 solution (2 × 3 mL) and saturated NaCl solution (2 × 3 mL). The organic layer was then dried over anhydrous Na_2SO_4 and filtered. The filtrate was concentrated under a vacuum to afford the corresponding crude product, which was purified by column chromatography to afford Biotin-InuA (5 mg, 18.5%).
